# The Role of Pocus in Acute Respiratory Failure: A Narrative Review on Airway and Breathing Assessment

**DOI:** 10.3390/jcm13030750

**Published:** 2024-01-28

**Authors:** Stefano Sartini, Lorenzo Ferrari, Ombretta Cutuli, Luca Castellani, Maddalena Bagnasco, Luca Moisio Corsello, Cristina Bracco, Maria Luisa Cristina, Eleonora Arboscello, Marina Sartini

**Affiliations:** 1 Emergency Medicine Department, IRCCS Policlinico San Martino, Largo Rosanna Benzi 10, 16132 Genoa, Italy; ombretta.cutuli@hsanmartino.it (O.C.); caste_luca@yahoo.it (L.C.); maddalena.bagnasco@hsanmartino.it (M.B.); luca.moisiocorsello@hsanmartino.it (L.M.C.); cristina.bracco@hsanmartino.it (C.B.); eleonora.arboscello@hsanmartino.it (E.A.); 2 Emergency Medicine Post-Graduate School, University of Genoa, Via Balbi 5, 16126 Genoa, Italy; lorfer@outlook.it; 3 Department of Health Sciences, University of Genoa, 16132 Genoa, Italy; sartini@unige.it; 4 Hospital Hygiene, E.O. Ospedali Galliera, Via Alessandro Volta 8, 16128 Genoa, Italy

**Keywords:** acute respiratory failure, lung ultrasound, upper airways management, pneumonia, pneumothorax, lung effusion, COPD, heart failure, diaphragm impairment, acute heart failure

## Abstract

Acute respiratory failure (ARF) is a challenging condition that clinicians, especially in emergency settings, have to face frequently. Especially in emergency settings, many underlying diseases can lead to ARF and life-threatening conditions have to be promptly assessed and correctly treated to avoid unfavorable outcomes. In recent years, point-of-care ultrasound (POCUS) gained growing consideration due to its bedside utilization, reliability and reproducibility even in emergency settings especially in unstable patients. Research on POCUS application to assess ARF has been largely reported mainly with observational studies showing heterogeneous results from many different applications. This narrative review describes the wide potentiality of POCUS to face airways and breathing life-threatening conditions such as upper airway management, pulmonary and pleural pathologies and diaphragm impairment. We conducted extensive research of the literature to report from major studies to case reports deemed useful in practical clinical utilization of POCUS in ARF. Due to the huge amount of the literature found, we focused on airways and breathing assessment trying to systematize the evidence according to clinical care of ARF in emergency settings. Further studies, possibly trials, should determine how POCUS is crucial in clinical practice in terms of standard of care improvements, patient safety and cost-benefit analysis.

## 1. Introduction

Acute respiratory failure (ARF) is a life-threatening condition characterized by acute onset of hypoxemia due to many clinical disorders such as pneumonia, congestive heart failure, aspiration and trauma [1,2]. Ventilation/perfusion mismatch and impaired excretion of carbon dioxide are the principal components of respiratory failure leading to systemic hypoxia and tissue damage [3]. It is essential to promptly identify the underlying cause to put in place a tailored treatment to correctly face the pathophysiological mechanism of injury.

Over the last decades, point-of-care ultrasound (POCUS) has gained increasing consideration and widespread utilization among emergency care settings due to its availability at bedside, reliability, reproducibility and overall cost-effectiveness [4]. Furthermore, it is an essential tool to assess acutely ill patients in low-resources fields such as prehospital settings and in those so unstable that radiological images are difficult to obtain [5,6].

The overwhelming application of POCUS in the assessment of ARF has been reported in many clinical scenarios and settings that vary from upper to lower respiratory airways, from cardiovascular to respiratory muscles analysis.

In many studies, POCUS showed a higher diagnostic accuracy compared to standard chest radiography to identify pulmonary edema, pneumothorax, lung effusion and pneumonia. Furthermore, good levels of accuracy are reported when compared to second-level imaging exams such as CT scans. Due to the extensive body of literature and data on this subject, a decision was made to undertake an exhaustive review of contemporary literature, in the form of a narrative review, with the objective of systematically organizing all available published data regarding the evaluation and management of acute respiratory failure using point-of-care ultrasound (POCUS).

## 2. Materials and Methods

A comprehensive search was conducted across the PubMed/Medline, Scopus, Cochrane and Google Scholar databases. The search incorporated the following search terms, utilizing Medical Subject Headings (MeSH) terms in adherence to the National Center for Biotechnology Information (NCBI) nomenclature and guidelines: “Lung AND ultrasound AND acute respiratory failure OR Pneumonia OR dyspnea OR edema OR heart failure OR Point of Care Ultrasound OR Major trauma”.

Inclusion criteria encompassed prospective and retrospective studies, case reports and case series published in either English or Italian, with no temporal constraints, exclusively focusing on patients in emergency departments (ED) or intensive care units (ICU). Publications concerning patients who had undergone surgery or had been admitted to geriatric wards were systematically excluded. Furthermore, we excluded COVID-19-related publications as they considered a specific topic, which was not the aim of this review.

The selected publications were categorized into five distinct groups:

A. Papers concerning upper airway or intubation;
B. Papers addressing respiratory function breathing-related impairment.

Two of the authors independently reviewed the literature. Articles were initially screened based on their titles and abstracts, employing the Rayyan platform for Systematic Review (https://www.rayyan.ai/). Subsequently, the full text of relevant research was acquired and rigorously assessed. Additionally, the references of the selected articles were scrutinized to ensure the comprehensive inclusion of relevant research. Any instances of disagreement were resolved through discussion until a consensus was achieved. 

We decided to report the research methodology of this narrative review to clarify the key points used and to explicit the typology of studies cited.

## 3. Results

A total of 1407 papers were initially selected, of which 1324 were excluded for not meeting the inclusion criteria. Subsequently, a state-of-the-art review was conducted to describe the current matters on the topic; thus, favorable and unfavorable results were included and discussed to obtain an overall view of the evidence reported in the literature.

## 4. Discussion

### 4.1. A: US & Airway

We listed the studies reviewed on the use of POCUS to assess and manage upper airways divided into sections: endotracheal tube positioning, upper airway damage identification, difficult airways management and post-trauma injury identification, laringeal edema assessment pre-extubation (Table 1).

Since recent years, ultrasound evaluation of upper and lower airways has been considered to have a marginal role. Over the last decade, the correlation between US airway findings and endoscopic examination (considered the gold standard) has been studied promoting its utilization in the clinical practice [18,19,20].

**Figure 1:**
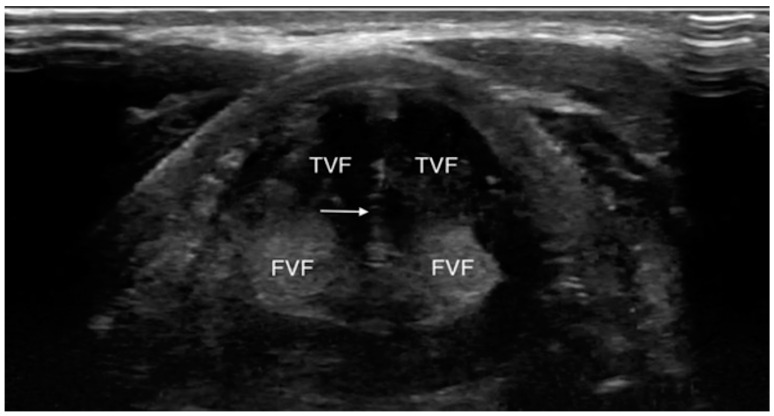
Transverse view of normal vocal folds during valsalva (adduction). FVF, false vocal fold; TVF, true vocal fold. White arrow indicates hyperechoic medial margins of true vocal folds meeting in midline. Reproduced with permission of Dr. Noel from [20].

**Figure 2:**
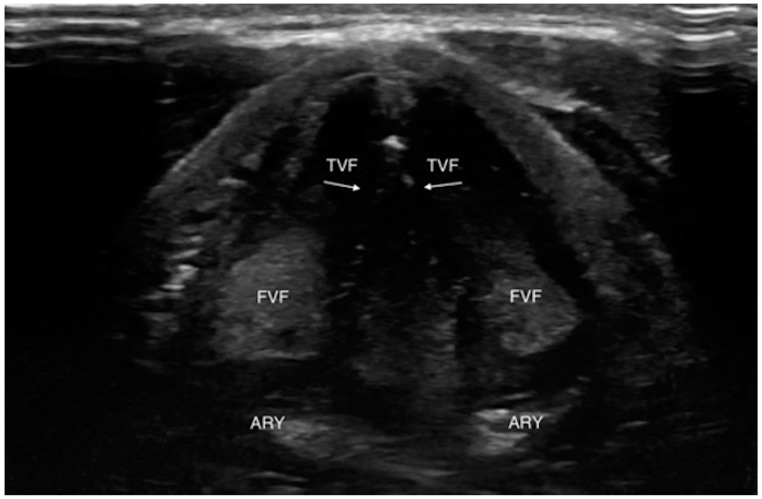
Transverse view of normal vocal folds during relaxation (abduction). ARY, arytenoid; FVF, false vocal fold; TVF, true vocal fold. White arrows indicate hyperechoic medial margins of true vocal folds. Reproduced with permission of Dr. Noel from [20].

Even if the upper airway US is affected by some limitations due to the presence of artifacts interfering with ultrasound images and interpretation such as subcutaneous emphysema, posterior laryngeal injury, cartilage calcification and foreign bodies, its utilization has been reported in many clinical scenarios [20] (Figure 1 and Figure 2).

#### 4.1.1. Endotracheal Tube (ETT) Positioning Assessment

The most common utilization of POCUS in upper airway assessment of patients with ARF reported in literature is the correct placement of the ETT in many studies and case reports that document US feasibility and reliability.

The incorrect positioning of the ETT expose critically ill patients at risk to develop severe complications and to secure a definitive airway. In clinical practice lung auscultation, End Tidal CO2 (ETCO2) and chest XR are commonly used to confirm ETT position, however US of airways and lung may also play a role.

US assessment of a correct intubation is based on the identification of the “double trachea sign” by the anterior neck approach. Firstly, this routine check was performed in the operating room, then applied in the emergency department. In this prospective observational study, operators performed bedside US within 3 min after patient arrival in ED or after endotracheal intubation. It was observed that sonographers achieved 100% accuracy with respect to determining the correct ETT position utilizing an anterior neck approach (while the intubators’ accuracy in assessing correct tube location was 97% compared to the clinical outcome). A blinded review of sonography findings confirmed all bedside US anatomical findings. An ultrasound-empty esophagus was 100% specific for endotracheal intubation, and a “double trachea sign” was 100% sensitive and 91% specific for esophageal intubation. The sonographic time to diagnosis was significantly faster than the intubator time to diagnosis (“easy” *p* < 0.001; *n* = 47; “moderate” *p* = 0.001; *n* = 15; “difficult” *p* < 0.001; *n* = 19) [7].

In this randomized controlled study, a group of residents scanned cadavers’ necks to confirm a correct endotracheal intubation or esophageal intubation. They were blinded to endotracheal tube placement and had to scan using either the B-mode method or B-mode plus color-Doppler. Moreover, a limited scanning time was given: 6 s for scanning with B-mode and 8 s for scanning with B-mode plus color-Doppler. There were 91.7% correct identifications made with B-mode and 86.9% with B-mode plus color-Doppler (*p*-value = 0.007). Finally, a correlation between the year of training and higher accuracy in ultrasound-guided ETT placement identification was observed [9].

In this prospective educational study by Chenkin et al., they tried to define the learning curve of POCUS to confirm ETT placement after a 10 min web-based tutorial and two practical sessions; participants were asked to correctly recognize the ETT position from ultrasound videoclips among a series of 20 endotracheal and 20 esophageal ETT placements. The study reported a sensitivity of 98.3% (95% CI 96.3–99.4%) and a specificity of 100% (95% CI 98.9–100%) for the participant to indicate the correct or incorrect placement [21]. Even if in this cadaveric-based study a superiority of POCUS to ETCO2 for ETT confirmation placement was found, we need to consider that capnography is less reliable mainly during cardiopulmonary resuscitation due to cardiovascular and metabolic impairment affecting CO2 delivery to the lung [8]. Considering that real-life studies are lacking on this matter, the evidence collected suggests that ETCO2 remains the first option, but POCUS may play a predominant role during cardiopulmonary resuscitation and in case of ETCO2 unavailability.

In addition, lung US can assess pleural sliding and thus can confirm lung aeration after intubation. In a monocentric observational study conducted at the Emergency Department of the National Taiwan University Hospital, 115 intubated patients were included. They were evaluated both with US and chest radiography as the gold standard method. The overall accuracy of bilateral sliding assessed by US was 88.7%, the positive predictive value was 94.7% (95% CI: 87.1–97.9%) in the non-cardiac-arrest group and 100% (95% CI: 87.1–100.0%) in the cardiac-arrest group. Furthermore, an advantage in time reduction was observed: the mean execution time of US bilateral sliding assessment was 88 s versus 1349 s of radiography [10].

#### 4.1.2. Upper Airways Damage Identification and Procedures

US visualization of upper airway structures and their abnormalities is a recent role of the POCUS application. Cases of laryngeal ACE-I-induced larynx edema [11] and trauma affecting the larynx have been approached with US to assess the extent of damage [12].

An important finding in upper airways US is cricoid membrane identification. US has been extensively used to guide invasive procedures and its role to support cricothyrotomy has been evaluated. In a prospective observational study emergency physicians applied a technique first learned in a cadaver laboratory and then applied in vivo. US did not affect the time of execution, as the mean time required was 24.32 s. This time was not affected by patient anatomy or body mass index (BMI) [13].

There are some case reports about patients presenting with a critical mass in the larynx. Upper airways US permitted to evaluate their extension and to identify the feasibility of cricothyroidotomy instead of emergency tracheostomy. In one of these cases, US was also applied to visualize the hyoid bone to assess short hyomental distance ratio, high pre-tracheal anterior neck thickness and tongue size to predict endotracheal tube size [14,15].

In emergency situations, POCUS appears crucial when a CT scan is not available or when patients are so unstable that they cannot leave the shock room (Table 2).

Upper airway US assessment is affected by some limitations mainly due to the presence of artifacts that may interfere with ultrasound images and interpretation such as subcutaneous emphysema, posterior laryngeal injury, cartilage calcification and foreign bodies [12].

#### 4.1.3. Laryngeal Edema Assessment Pre-Extubation

The assessment of the larynx is important to predict extubation failure. Usually, this is evaluated by a leak test (difference between expiratory tidal volumes with the cuff inflated and deflated). Two prospective observational studies evaluated the air column width differences (ACWD) (width of air between the vocal cords seen by laryngeal ultrasonography) as a predictive index of extubation failure. In the first study ACWD ≥ 1.6 mm predicted laryngeal edema with 0.706 and 0.702 sensitivity and specificity, respectively; the area under the receiver operating characteristic curve of laryngeal ultrasound was 0.823 (95% confidence interval, 0.698–0.947) and that of cuff leak test was 0.840 [19]. In the other study, both laryngeal US and leak test resulted in having a positive predictive value < 20% cuff leak test (cut-off point: 249 mL) and showed a sensitivity and specificity of 75% and 59%, respectively. In addition, laryngeal ultrasonography (cut-off point for air column width: 10.95 mm) resulted in a sensitivity and specificity of 50% and 54%, respectively [17].

A meta-analysis aimed to assess the diagnostic accuracy of ACWD to predict post-extubation stridor analyzed observational studies finding, for a cut-off value from 0.45 to 1.6 mm, a pooled sensitivity and specificity of 0.8 (95% CI = 0.69–0.88, *I*^2^: 37.26%, eight studies) and 0.81 (95% CI = 0.72–0.88, *I*^2^: 89.51%, eight studies), respectively, and a pooled AUC of 0.87 (95% CI = 0.84–0.90). However, they concluded that the strength of the evidence was poor [22]. From these non-univocal results highlighted by the variability of the reported cut-off, we may argue that the ACWD is a challenging measurement to obtain influenced by operator experience, method standardization and the confounding presence of many artifacts. Further prospective studies are needed to assess its real potential in clinical practice.

**Figure 3:**
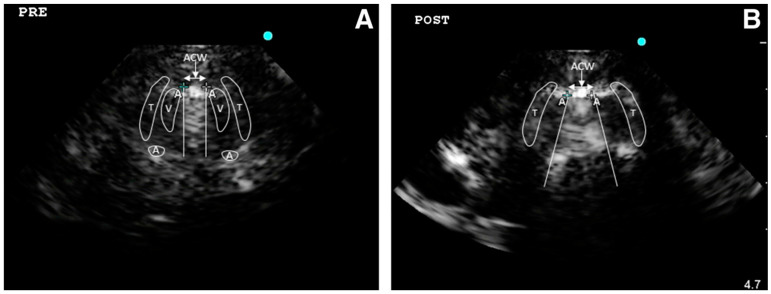
The imaging from laryngeal ultrasound demonstrates the air column width before (**A**) and after (**B**) endotracheal tube cuff deflation. After cuff deflation, the fan-shaped widening air column width obliterates surrounding structures such as vocal cords and arytenoid cartilage by acoustic shadow (T, thyroid cartilage; V, vocal cord; A, arytenoid cartilage). Reproduced with permission of Dr. Sutherasan from [16].

Even if the literature on airway US application is still relatively limited, its utilization in clinical practice, both in pre-hospital and emergency hospital settings, is increasing and US can be considered a useful tool to support physicians in airway management especially when they are difficult (Figure 3).

### 4.2. B: US and Breathing 

We reported the studies reviewed on the use of POCUS to assess and manage breathing impairments. They are mainly observational and sistematic review, only three are randomised controlled trial (Table 3).

#### 4.2.1. Protocols on Lung US

**Figure 4:**
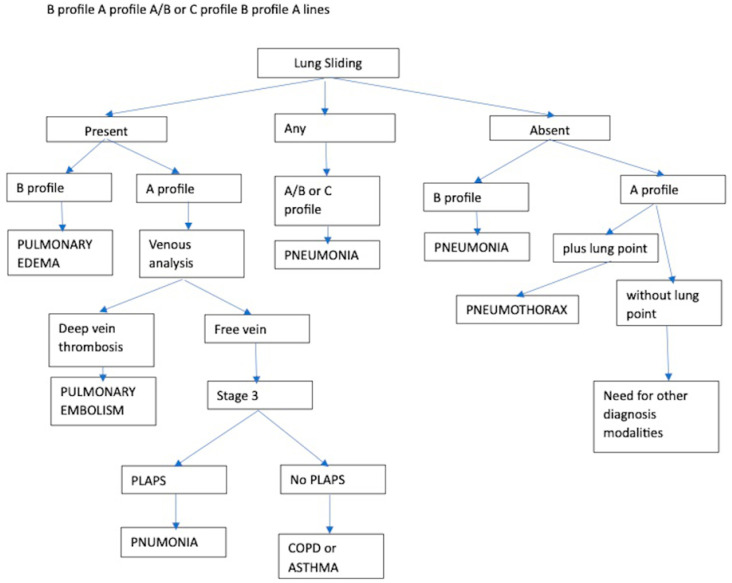
The Blue Protocol ([80], modified).

The BLUE protocol (Figure 4) is a flow chart to approach acute respiratory failure and its differential diagnosis by lung US (LUS) in a standardized way proposed by Lichtenstein and updated in 2008 [23].

It is based on the identification and interpretation of easy LUS findings indicating normal lung surface (bat sign, lung sliding, A-lines), pleural effusions (quad and sinusoid sign), lung consolidations (fractal and tissue-like sign), interstitial syndrome (lung rockets or B lines), pneumothorax (stratosphere sign and the lung point) and venous thrombosis (compressive venous ultrasound) [80].

Mastering this basic echographic semiology allows clinicians to seek after the principal differential diagnosis affecting pulmonary parenchyma and pleural space: pneumonia, hemodynamic pulmonary edema, exacerbated chronic obstructive pulmonary disease or asthma, pulmonary embolism or pneumothorax [81].

In a meta-analysis by Asmara et al. in which 4 studies were analyzed for a total of 820 patients, BLUE protocol showed a sensitivity of 84% (76–89%), a specificity of 98% (93–99%), LR+ 42 (12–147), LR− 0.12 (0.07–0.2) and odds ratio 252 (81–788) for pneumonia and sensitivity 89% (95% CI, 81–93%), specificity 94% (89–96%), LR+ 14 (8–25), LR− 0.165 (0.11–0.24), and DOR 116 (42–320) for pulmonary edema [24].

Prospective observational studies have been conducted to verify sensitivity and specificity, comparing diagnoses made by BLUE and final discharge diagnoses. In two articles that evaluated 37 and 50 patients respectively, pneumonia and pulmonary edema were diagnosed with sensitivities of 88–94% and 86–92% and specificities of 90–94% and 87–100%, respectively [25,26]. A similar study enrolled 130 patients to measure BLUE protocol diagnostic accuracy in different diseases, it was as follows: 95.38% for pulmonary edema, 100% for pneumothorax, 93.85% for pneumonia, 96.92% for chronic obstructive pulmonary disease, 99.23% for pulmonary thromboembolism and 95.38% for acute respiratory distress syndrome [27].

However, some evidence suggests that the BLUE protocol did not always reach a correct diagnosis. A case of a pregnant woman affected by the rupture of a pulmonary hydatid cyst and a case of pulmonary hemorrhage not identified by BLUE protocol have been reported [28,29].

Indeed, BLUE is not the only protocol proposed to assess causes of respiratory failure. FALLS is a development of BLUE protocol based on initial basic cardiac sonography assessment, that allows to sequentially rule out obstructive, then cardiogenic, then hypovolemic shock for expediting the diagnosis of distributive (usually septic) shock [82].

Specifically for pneumonia, ultrasonographic findings are subpleural consolidations, positive air bronchogram within an echo-poor area and basal effusion [30,83]. 

Overall sensitivity and specificity reported by two meta-analyses were 85–94% and 93–96%, positive and negative LRs were 11.05–16.8 and 0.07–0.08 and the area under the ROC was 0.98–0.99 [31,32].

Case reports about pneumonia highlighted how US helps the clinicians to reach the correct diagnosis, also in case of extreme presentation, such as in a case of massive pleural parapneumonic effusion that could not be characterized by chest radiography [33,34].

Pneumothorax ultrasound echographic diagnosis is deeply accepted and supported by evidence. Different findings can identify PNX:

- Abolition of lung sliding alone, sensitivity 100% specificity 78%;
- Absent lung sliding plus the A-line sign, sensitivity 95% specificity 94%;
- Lung point, sensitivity 79% specificity 100% [35].

While LUS demonstrated good reliability in diagnosing PNX and it is easily deployable by emergency physicians in clinical practice [36], clinicians should exercise caution and always correlate clinical presentation to avoid misinterpretation of US findings. Other factors, such as hypercapnia and BMI in patients with COPD and asthma, as well as pneumothorax or pleural talcage, can contribute to the loss of lung sliding [37]. In a case report describing an acute exacerbation of COPD, a patient presented a large bulla that mimicked a lung point to POCUS without an actual PNX [38].

##### Acute Respiratory Distress Syndrome (ARDS)

A particular mention to ARDS, US has been investigated for diagnostic and monitorization purposes, as it can predict CT findings about lung aeration, monitor lung re-aeration during treatment and identify tidal volume recruitment [84].

Many prospective observational studies evaluated lung ultrasound scores in ARDS. Firstly, the combination of LUS plus pulse oximetry showed a better diagnostic accuracy than chest radiography plus blood gas analysis [39]. Moreover, LUS correlated well with oxygenation (P/F ratio) and seems to have a prognostic value about survival in mechanically ventilated patients and post-extubation distress syndrome [40,41,42,43].

However, in a retrospective observational study comparing chest radiography versus LUS to diagnose ARDS according to Berlin’s definition, even if the latter was more reliable in assessing the severity of the disease, chest radiography showed to be complementary (more than interchangeable) to LUS [44].

**Figure 5:**
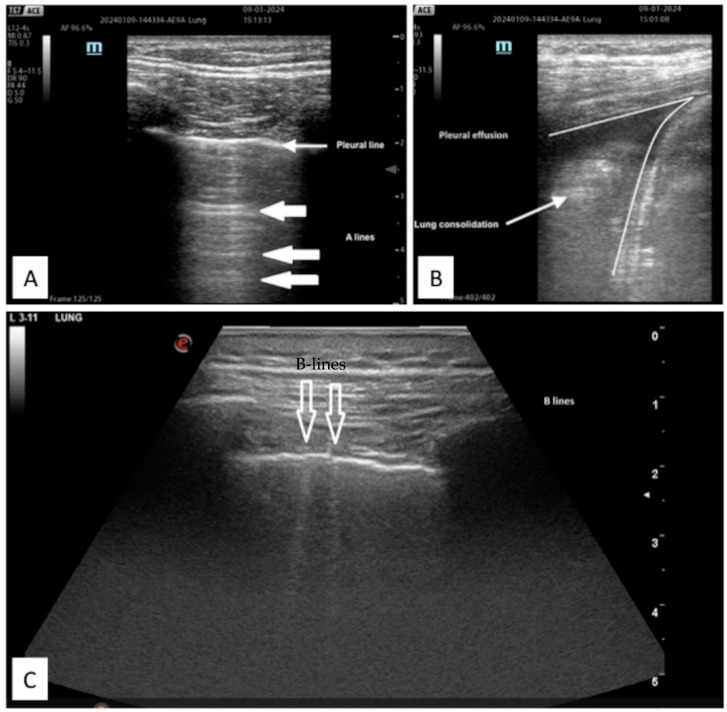
Example of LUS images of (**A**) normal aerated lung with pleural and A-line; (**B**) pleural effusion and associated parenchymal consolidation; (**C**) B-lines sign of interstitial syndrome.

LUS application in ARDS assessment has been so extensively studied that a possible correlation with a genetic polymorphism in the plasma platelet-activating factor was published. G994T polymorphism, combined with LUS score, showed a negative correlation with respiratory failure index, the need for ventilation, lactic acid levels, SOFA score, etc. A combination of LUS score and G994T polymorphism may be employed as a potential prognostic marker for ARDS [45] (Figure 5).

#### 4.2.2. Diagnostic Accuracy

Many studies compared the POCUS approach to standard clinical care in emergency settings to analyze possible advantages in terms of diagnostic accuracy, appropriate treatment given and shortening management time.

We found four studies that compared the US approach to standard clinical evaluation (Table 4) [46,47,48,49].

In a prospective controlled blinded study, POCUS application searching for life-threatening conditions in critically ill patients showed a sensitivity of 100%, specificity of 93.3%, positive predictive value of 76.7% and negative predictive value of 100% [49].

In this prospective observational study conducted by Barman et al., POCUS was performed after a first clinical diagnosis was made. Out of 108 enrolled patients, initial clinical diagnosis was appropriate in 67.5% of cases, after POCUS assessment the diagnostic accuracy raised to 88% adding or changing the diagnosis in 37% of cases. Similar improvements were observed in the treatment plan decided before and after POCUS, in 36% of cases treatment decisions were changed. This study highlights how POCUS can improve diagnostic accuracy and lead to different treatment choices in clinical practice [50].

Furthermore, in this interventional study by Sen et al. about the medical emergency team activities, POCUS was proved to be feasible and reliable for in-hospital emergency management [51].

Diagnostic accuracy has also been evaluated for single pathology. The accuracy of LUS in a prospective observational study [52] is reported below:

- Pneumonia, standard, 0.74, ultrasound, 0.87;
- Acute hemodynamic pulmonary edema standard, 0.79, ultrasound, 0.93);
- Decompensated COPD standard, 0.8, ultrasound, 0.92;
- Pulmonary embolism standard, 0.65, ultrasound, 0.81;

We found two meta-analyses [53,54] that report sensitivity and specificity for the following diagnosis comparing the standard of care to US:

- Pneumonia/consolidation 89–92% and 94–97%;
- Heart failure/interstitial syndrome 90–95% and 91–93%;
- Pleural effusion 95% and 99%;
- COPD/asthma (A profile) 78% and 94%.

POCUS diagnostic accuracy has also been assessed comparing specifically LUS to CT scan findings in ARF patients. Overall, LUS sensitivity and specificity were 82.7–92.3% and 90.2–98.6% reaching a global agreement with CT scans ranging from 0.640 (0.391–0.889) to 0.934 (0.605–1.000) with an average of 0.775 (0.577–0.973) [53]. In another review, LUS is reported to reach a specificity between 78 and 100%, when compared to CT [56].

In a prospective comparative study for a specific diagnosis, sensitivity and specificity between LUS and CT lung scan were evaluated: consolidations 76–83% versus 92–95.5%, interstitial syndrome 60% versus 69%, PNX 59% versus 97%, pleural effusion 85% versus 77%, respectively [51]. Moreover, in this prospective cohort study made by Tierney et al. [57], the agreement for localization of pathology was assessed. It showed that LUS correctly localized pathology with an 83.6–96% agreement with chest CT scans. 

Moreover, many studies showed a significant superiority of LUS with respect to chest plain XR accuracy to identify lung consolidation (sensitivity 81.4% vs. 64.3%) [58].

#### 4.2.3. Time-to-Diagnosis Improvement 

POCUS turned out to be useful also in shortening the time to reach a diagnosis and reducing patient overall management. One of Lichtenstein’s works on US is reported to save up to two hours for diagnosis and management [23].

The mean time for diagnosis was shorter in POCUS application versus standard care. In these two prospective observational studies and a prospective randomized study, the time needed for a diagnosis was 12–42 min with POCUS, against 79–270 min with usual clinical care [59,60,61]. 

Similarly, in a prospective multicenter superiority trial made in Danish Emergency Departments by Riishede et al. [46], a reduction in overall hospital staying was found in the POCUS group versus usual clinical care.

#### 4.2.4. Diaphragm Ultra-Sound (DUS)

Other than lung ultrasound, the evaluation of the diaphragm in ARF has been studied to assess the entity of respiratory distress. Even if DUS is not precisely standardized yet [85], with US is possible to obtain information about diaphragm movement.

As the diaphragm is the most important inspiratory muscle, its dysfunction has a great impact on the deterioration of respiratory function. Indeed, literature about DUS has been focused on its predictive value: prediction of respiratory failure, NIV failure and weaning/extubation failure [62]. Furthermore, DUS application was reported to document diaphragmatic dysfunction in a case of dermatomyositis [63].

**Figure 6:**
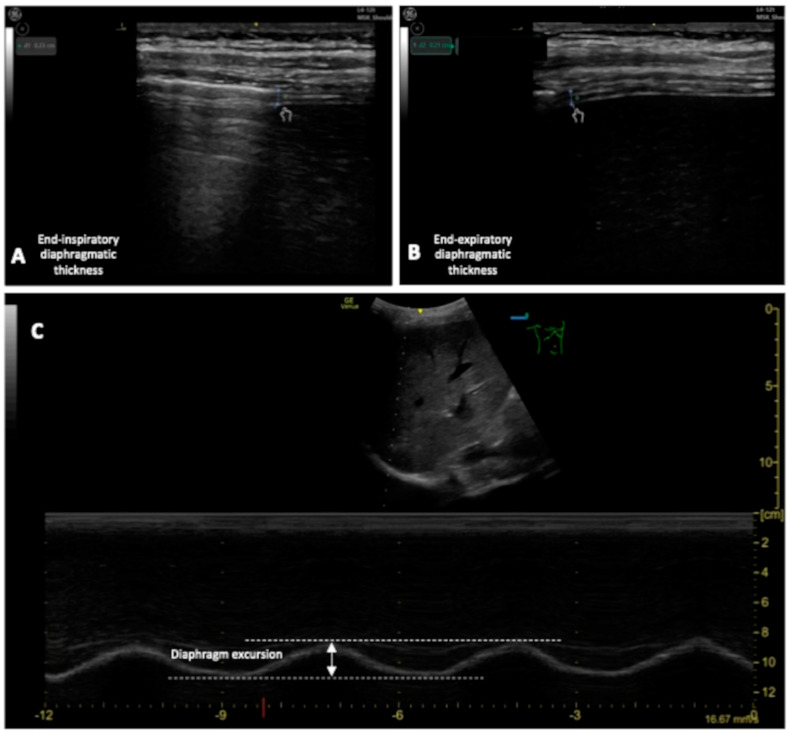
(**A**,**B**): Measurement of the end-inspiratory and end-expiratory diaphragmatic thickness; (**C**) measurement of the DE.

To quantify diaphragm movement by DUS clinicians can measure (see Figure 6):

- Diaphragm thickening fraction (DTF), measurement of the difference in end-inspiratory and end-expiratory diaphragmatic thickness, expressed as a fraction;
- Diaphragm excursion (DE), the diaphragmatic altitude difference between expiration and inspiration [86] (Figure 6).

No significant differences in measurement made by M or B mode were found [64].

The main limitations of the technique are as follows: diaphragm excursion varies with BMI, movements of the organs continuous to the diaphragm are not reliable to estimate diaphragm movements, in intercostal insonation lung may obstacles tidal evaluation of the diaphragm and in subcostal positioning of the probe may diminish lung interposition but US incidence angle may affect measuring precision [86]. 

DTF reduction proved to be a reliable tool to assess the risk of respiratory failure in patients affected by pneumonia. (The optimal DTF cut-off was 23.95%, with an OR: 0.939, *p* = 0.0416, 69.23% of sensitivity, 83.78% of specificity, 88.57% of negative predictive value and 80% of accuracy) [65].

Studies about COPD focus on the prediction of NIV failure. They found that diaphragm ultrasound showed great potential to evaluate diaphragm function, especially to assess changes in diaphragmatic function in patients with stable COPD and to predict the success rate of NIV and MV weaning in patients with acute exacerbation [87]. Many studies found that diaphragm dysfunction correlates to NIV failure (and with corticosteroid therapy, prolonged MV and tracheostomy) [66]. DTF with a cut-off value of <26–29% on both hemidiaphragm was able to predict NIV failure in acute exacerbation of COPD, with 96.67% sensitivity and 80–82.22% specificity [67]. In another similar study, DTF < 20% was the cut-off to identify diaphragm dysfunction with an AUC of 0.84. And it showed to be a better predictor than blood starting pH and pCO2 values [68].

Furthermore, even DE correlated to NIV success or failure. In a prospective observational study, DE values were significantly higher in NIV successes group than that in NIV failure group (T0 (1.92 [1.22–2.54] cm versus 1.00 [0.60–1.41] cm, *p* = 0.02), at T1 (2.14 [1.76–2.77] cm versus 0.93 [0.82–1.27] cm, *p* = 0.007), and at T2 (1.99 [1.63–2.54] cm versus 1.20 [0.79–1.41] cm, *p* = 0.008), respectively) [69]. 

However, another prospective observational study did not document a significant correlation between DUS and diaphragm dysfunction. Sensitivity and specificity of the DE for NIV failure were 58.1% and 62.5%, respectively [70]. 

DUS was employed also in a comparative study to assess diaphragm work during different techniques of respiratory support therapies [71]. 

Spontaneous breathing trials are commonly performed before extubation to predict post-extubation NIV or risk of reintubation. DUS may contribute to assessing this prediction [72].

In a cross-sectional comparative study, a DE cut-off of 1.2 cm showed a sensitivity and specificity for successful weaning of 78.95% and 70.83%, respectively [73]. In other prospective studies, DTF sensitivity and specificity for successful weaning were 93–96% and 58–68%, while (in one of these two studies) LUS showed to be less sensitive but more specific (76% and 73%) [74,75].

One case report talks about a typical extubation failure assessed with DUS probably due to diaphragm dysfunction in a case of septic shock due to pneumonia [62]. 

A prospective observational study found a sensitivity and specificity of a holistic approach to predict extubation failure in mechanically ventilated patients for 72 h or more, of 100% (78.2–100%) and 7.7% (2.5–17.1%), respectively, with an AUC of 0.54. The sensitivity and specificity of diaphragm thickening fraction, using a cut-off value of <30% for extubation failure were 86.7% (59.5–98.3%) and 25.4% (15.5–37.5%), respectively, with an AUC of 0.61 [76].

As mentioned before, DUS may also assume a diagnostic role in evaluating respiratory failure due to neuro-muscular conditions. We found four case reports describing the identification of hemidiaphragmatic dysfunction with DUS. The underlying causes were phrenic nerve paralysis, dermatomyositis exacerbation, brachial plexus damage and amyotrophic lateral sclerosis, but diaphragm dysfunction assessment was the main finding that led to the correct diagnosis [63,77,78,79].

## 5. Limitations

POCUS application in clinical practice and its effectiveness in reducing unfavorable outcomes are affected by some limitations from many different points of view:

- Availability and settings: the lack of ultrasound machines in specific settings like pre-hospital or in limited-resource countries; furthermore, updated software and probes are needed to obtain more reliable images. Moreover, in case of intensive use from patient to patient and the lack of disinfection and cleanliness, the probes could be a vector of infection [88].
- Technical impairment: “air” in itself is a limitation to ultrasonic wave propagation and their interaction with body tissue and fluids generates artifacts that have to be recognized and correctly interpreted. Furthermore, the correct use of the different probes and the many settings allowed by the new ultrasound machine is mandatory to properly set up adequate images. Finally, the lack of standardization with specific protocols for upper and lower airway POCUS execution may limit replication and increase interobserver variability [89].
- Competences: education in the POCUS technique and an adequate level of experience are cardinal points to obtain a reliable POCUS assessment. Continuing US utilization in daily clinical practice, comparison with other gold standard imaging exams and support of senior team members are needed to avoid clinical errors and to improve personal skills. Specific ultrasound training programs should be implemented in the trainee core curriculum [90].
- Scientific level evidence: most of the published data about POCUS clinical utilization and effectiveness are based on observational studies. However, it is difficult to plan studies with strong levels of evidence such as TRIAL or prospective multicentric and interventional studies due to organizational and methodological impairments such as different ultrasound machines in different settings, interobserver variability, availability of ultrasonologists with the same level of competencies, contradictory outcomes identification and measurements.

Even in light of such considerations, POCUS is undoubtedly a useful clinical tool, and further and stronger evidence is needed to fully support its utilization.

## 6. Conclusions

POCUS application to assess ARF is becoming a useful and reliable tool, especially in emergency settings supported by growing scientific evidence. The availability of an ultrasound machine in increasing settings allows its application in many different clinical conditions, thus its utilization should be implemented and reported to increase literature evidence on its potentiality.

Emergency medicine is one of the main disciplines where POCUS may make the difference between life and death being useful also in procedural intervention guidance if needed. Individual competence, poor resources and adverse environmental conditions may limit its application; however, updated and new ultrasound technology may help clinicians to fill these gaps. Unfortunately, the grade of scientific evidence on POCUS such as clinical trials is poor, and its increasing utilization should lead to conducting studies with a stronger level of evidence such as clinical trials.

## Figures and Tables

**Table 1:** US and airway studies.

First Author, Year	State	Kind of Study	N. Subject
*Endotracheal tube (ETT) positioning assessment*			
Hoffmann B et al., 2014 [7]	USA	Observational study	86
Wojtczak JA et al., 2014 [8]	USA	Sperimental	
Hossein-Nejad, 2021 [9]	Iran	RCT	16 students perform trial on 3 different cadavers
Sim SS et al., 2011 [10]	Taiwan	Observational study	115
*Upper Airways damage identification*			
Schick M et al., 2016 [11]	USA	Case report	1
Adi O et al., 2020 [12]	Malaysia	Case series	4
*Difficult airways management and post-trauma injury identification*			
Nicholls SE et al., 2008 [13]	USA	Quasi sperimental	50
Adi O et al., 2021 [14]	Malaysia	Case report	1
Iqhbal M et al., 2018 [15]	Malaysia	Case report	1
*Laringeal edema assessment pre-extubation*			
Sutherasan Y et al., 2013 [16]	Thailand	Observational study	101
Mikaeili H 2014 [17]	Iran	Prospective study	41

**Table 2:** Proposed focused airway ultrasound findings in correlation to the Schaefer Classification System and standard management of laryngeal injury (Adi O et al. [12], 2020; modified).

Schaefer Classification	CT Scan Findings, Based on Schaefer Classification	Focused Airway Ultrasound Findings	Standard Management and Intervention
Group 1	Minor endolaryngeal hematoma or laceration without detectable fracture	Endolaryngeal hematoma without detectable fracture	Supportive care including observation, antibiotics, humidified air, supplemental oxygen, anti-reflux medications, voice rest and early steroid administration
Group 2	Edema, hematoma, minor mucosal disruption without exposed cartilage, nondisplaced fracture noted on CT	Edema, endolaryngeal hematoma, minor mucosal disruption without exposed cartilage, nondisplaced fracture, mucosal hematoma/edema, nondisplaced fracture of cartilage framework	Patients with Group 2 injuries should be serially examined, since the injuries may worsen or progress with time. Occasionally group 2 injuries may require a tracheotomy
Group 3	Massive edema, mucosal tear, exposed cartilage, cord immobility, displaced fracture	Edema, cord immobility, displaced fracture, vocal fold immobility, obvious displaced fracture	Direct laryngoscopy, esophagoscopy and immediate open surgical repair are deemed necessary due to extension of injuries
Group 4	Addition of more than two fracture lines or massive trauma to laryngeal mucosa	Addition of more than two fracture lines, comminuted fracture of laryngeal cartilage framework	
Group 5	Complete laryngeal separation		

**Table 3:** US and breathing studies.

First Author, Year	State	Kind of Study	N. Subject
*Protocol on lung US*			
Lichtenstein DA and Mezier GA, 2008 [23]	France	Observational study	301
Asmara OD et al., 2022 [24]	Indonesia	Systematic review and meta-analysis	
Dexheimer Neto FL et al., 2015 [25]	Brazil	Observational study	42
Patel CJ et al., 2018 [26]	India	Observational study	50
Chaitra S and Hattiholi VV, 2022 [27]	India	Cross-sectional study	130
Arslan B and Sonmez O, 2022 [28]	Instanbul	Case report	1
Haaksma, ME et al., 2019 [29]	The Netherlands	Case report	1
Staub LJ et al., 2019 [30]	Brazil	Systematic review and meta-analysis	
Chavez MA et al., 2014 [31]	Perù	Systematic review and meta-analysis	
Alzahrani, S.A [32]	Saudi Arabia	Sistematic review	
Grabala J et al., 2020 [33]	Poland	Case study	1
Gardecki J et al., 2019 [34]	USA	Case study	1
*Pneumothorax (PNX)*			
Lichtenstein DA et al., 2005 [35]	France	Observational study	200
Zhang G et al., 2021 [36]	China	Case report	1
Mallow C et al., 2019 [37]	USA	Observational study	159
Aziz SG et al., 2016 [38]	USA	Case report	1
*ARDS*			
Bass CM et al., 2015 [39]	USA	Prospective comparative study	77
Todur P et al., 2021 [40]	India	Observational study	37
Zhao Z et al., 2015 [41]	China	Observational study	21
Xie Y et al., 2021 [42]	China	Prospective observational study	121
Wang R et al., 2022 [43]	China	Prospective observational study	92
See KC et al., 2018 [44]	Singapore	Retrospective observational study	456
Lv W et al., 2019 [45]	China	Prospective observational (?)	112
*Diagnostic accuracy*			
Riishede M et al., 2021 [46]	Denmark	RCT	211
Mantuani D et al., 2016 [47]	UK	Observational study	57
Laursen CB et al., 2013 [48]	Denmark	Observational study	139
Zieleskiewicz L et al., 2013 [49]	France	Observational study	165
Barman B et al., 2020 [50]	India	Observational study	108
Sen S et al., 2017 [51]	USA	Prospective study	50
Silva S et al., 2013 [52]	France	Observational study	78
Yuan X et al., 2021 [53]	China	Systematic review and meta-analysis	
Smit JM et al., 2021 [54]	The Netherlands	Observational study	87
Chiumello et al., 2019 [55]	Italy	ERS statement	
Hew M et al., 2015 [56]	Singapore	Systematic review	
Tierney DM et al., 2020 [57]	USA	Cohort study	67
Nazerian P et al., 2015 [58]	Italy	Observational study	285
*Time-to-diagnosis improvement*			
Lichtenstein DA and Mezier GA, 2008 [23]	France	Observational study	301
Gaber HR et al., 2019 [59]	Egypt/USA	RCT	59
Zare MA et al., 2022 [60]	Iran	Observational study	103
Baid H et al., 2022 [61]	India	Observational study	237
Riishede M et al., 2021 [46]	Denmark	RCT	211
Kilaru D et al., 2021 [62]	USA	Case report	1
Chong WH et al., 2021 [63]	USA	Case report	1
Kalın BS et al., 2020 [64]	Turkey	Observational study	62
Chu SE et al., 2022 [65]	Taiwan	Cohort study	50
Antenora F et al., 2017 [66]	Italy	Pilot study	41
Elsayed AA et al., 2022 [67]	Canada	Observational study	15
Marchioni A et al., 2018 [68]	Italy	Cohort study	75
Cammarota G et al., 2019 [69]	Italy	Evaluation study	22
Barbariol F et al., 2021 [70]	Italy	Observational study	47
Laverdure F et al., 2019 [71]	France	Clinical trial	50
Shrestha GS et al., 2017 [72]	Nepal	Letter to editor	
Hayat A et al., 2017 [73]	UK	Cross-sectional comparative study	100
Pirompanich P and Romsaiyut S, 2018 [74]	Thailand	Observational study	34
Tenza-Lozano E et al., 2018 [75]	Spain	Cross-sectional comparative study	109
Haaksma ME et al., 2021 [76]	UK	Case report	1
Doyle MP et al., 2020 [77]	USA	Case report	1
Yajima W et al., 2022 [78]	Japan	Case report	1
Shrestha GS et al., 2014 [79]	Nepal	Case report	2

**Table 4:** Studies that compared the US approach to standard clinical evaluation.

		Diagnostic Accuracy with Standard Care vs. POCUS	Appropriate Treatment with Standard Care vs. POCUS
Controlled multicenter study	Riishede M et al., 2021 [46]	77.1–79.3%	65.7–79.3%
Observational study	Mantuani D et al., 2016 [47]	53–77%	
Randomized controlled study	Laursen CB et al., 2014 [48]	63.7–88.0%	
Prospective observational study	Zieleskiewicz L et al., 2013 [49]	80–94%	
